# Research Interest and Public Interest in Melanoma: A Bibliometric and Google Trends Analysis

**DOI:** 10.3389/fonc.2021.629687

**Published:** 2021-02-18

**Authors:** Hanlin Zhang, Yuanzhuo Wang, Qingyue Zheng, Keyun Tang, Rouyu Fang, Yuchen Wang, Qiuning Sun

**Affiliations:** Department of Dermatology, Peking Union Medical College Hospital, Chinese Academy of Medical Sciences, Peking Union Medical College, Beijing, China

**Keywords:** melanoma, bibliometric analysis, Google Trends, research interest, public interest

## Abstract

**Introduction:**

Melanoma is a severe skin cancer that metastasizes quickly. Bibliometric analysis can quantify hotspots of research interest. Google Trends can provide information to address public concerns.

**Methods:**

The top 15 most frequently cited articles on melanoma each year from 2015 to 2019, according to annual citations, were retrieved from the Web of Science database. Original articles, reviews, and research letters were included in this research. For the Google Trends analysis, the topic “Melanoma” was selected as the keyword. Online search data from 2004 to 2019 were collected. Four countries (New Zealand, Australia, the United States and the United Kingdom) were selected for seasonal analysis. Annual trends in relative search volume and seasonal variation were analyzed, and the top related topics and rising related topics were also selected and analyzed.

**Results:**

The top 15 most frequently cited articles each year were all original articles that focused on immunotherapy (n=8), omics (n=5), and the microbiome (n=2). The average relative search volume remained relatively stable across the years. The seasonal variation analysis revealed that the peak appeared in summer, and the valley appeared in winter. The diseases associated with or manifestations of melanoma, treatment options, risk factors, diagnostic tools, and prognosis were the topics in which the public was most interested. Most of the topics revealed by bibliometric and Google Trends analyses were consistent, with the exception of issues related to the molecular biology of melanoma.

**Conclusion:**

This study revealed the trends in research interest and public interest in melanoma, which may pave the way for further research.

## Introduction

Melanoma is a severe skin cancer that metastasizes quickly. Cutaneous melanoma causes 55,000 deaths each year, and once the disease spreads, it rapidly becomes life-threatening (1). Cases of cutaneous melanoma account for approximately 1.7% of all newly diagnosed cases of primary malignant cancers (1). The incidence and mortality rate of melanoma vary around the world. Fair-skinned populations are particularly prone to melanoma, and the incidence of melanoma is the highest in New Zealand and Australia (2). Exposure to ultraviolet radiation, number of atypical moles, and genetic background are common risk factors for melanoma (3).

Bibliometric analysis is a method used to quantify hot topics and research interest in the research community (4–6). Bibliometric analysis can provide physicians and investigators with crucial messages in a specific field. A thorough bibliometric analysis of the most frequently cited articles may facilitate an understanding of disciplinary development and future directions of a research field (7, 8). Google Trends is a commonly used tool for addressing online health issues. Infodemiological methods using Google Trends can estimate the epidemiological characteristics, explore the public interest, and monitor the dynamic variations in infectious diseases (9). Previously, some studies demonstrated positive correlations between the online search frequency of “melanoma” and that of its risk factors (10–12). However, McDonald and Bloom reported negative results on the association between the search index and the incidence of melanoma (13, 14).

Compared to bibliometric analysis, which provides information on research interest, Google Trends analysis provides information on public interest. Physicians and investigators should know not only the hotspots of scientific research on melanoma but also the issues of interest for the general public. This study aimed to update the topics of research interest and public interest in melanoma using bibliometric and Google Trends analyses and compare the similarities and differences, which may pave the way for further research.

## Methods

### Bibliometric Analysis

We analyzed the top 15 most frequently cited articles on melanoma each year from 2015 to 2019 according to the bibliometric analysis method. These publications were retrieved from the Web of Science in descending order according to their numbers of annual citations. Two researchers (H. Zhang and Y. Wang) independently screened the abstracts and reached a consensus on the qualifying papers. Articles focusing on multiple diseases, conference articles, patents, comments, or case reports were all excluded. Original articles, reviews, and research letters were all included in this research.

### Search Tool and Keyword Selection

Online search data were collected from Google Trends. Google Trends provided an index, namely, the relative search volume (RSV), to facilitate comparisons between terms, times, and locations. The RSV was restricted to a range from 0 to 100. An RSV of 100 represented the highest search count in a given period (weeks, months, or years), and the search counts were proportionally assigned lower numbers in other periods. For example, an RSV of 50 indicates that half as many searches were performed in the selected period compared to the searches indicated by an RSV of 100 (15). An RSV of 0 did not necessarily indicate 0 searches but may represent an extremely low search count compared to other periods (16). Google Trends also automatically adjusted the RSV based on population sizes to allow a comparison between populated areas and underpopulated areas (17).

The keywords were selected under the instruction of a previous guideline (18). Words or short phrases that were specific and not prone to be confused with other words or short phrases were preferable. Google Trends provided two types of query modes. One mode was the “Terms,” which could be combined for exhaustive search, but the results would only be shown in the given language. The other type was “Topics,” which were defined as groups of terms that shared the same concept in any language. This mode also included related searches in non-English speaking countries and might contain the most associated information (16). The mesh words of PubMed only provided “melanomas” for possible synonyms or homonyms of “melanoma” and allowed us to compare the two types of query modes by inputting different patterns of keywords, including “melanoma” alone as a term or topic, “melanomas” alone as a term, and “melanoma + melanomas” as a combination of terms in Google Trends. Both tests yielded similar fluctuations and patterns, but the topic “melanoma” produced the highest RSV. Therefore, the topic “Melanoma” was selected as the keyword in this study.

### Data Query

The “Health” category was chosen to exclude unrelated information. The time range was set from January 2004 to December 2019. On 1 September 2020, the RSV data were exported to Microsoft Excel 2019. Four English-speaking countries with high RSVs were selected for seasonal variation analysis. Two countries (the United Kingdom and the United States) were located in the Northern Hemisphere, and the other two countries (Australia and New Zealand) were located in the Southern Hemisphere.

### Google Trends Analysis

Topics related to the search term were also extracted from Google Trends to analyze the public interest. Google Trends provided two types of related topics: “Top related topics” and “Rising related topics.” “Top related topics” are defined as the most frequently searched topics within the chosen category, time, or country. “Rising related topics” are topics with high RSV growth and are presented as a percentage of fold changes. We queried the “Top related topics” and “Rising related topics” each year from 2014 to 2019 globally to analyze the variation in the public interest over time. The results were manually examined by two searchers (H. Zhang and Y. Wang) to exclude irrelevant information.

### Statistical Analysis

R software (v 3.6.2) was used for statistical analysis and plotting graphs. A diagram was plotted using the “plot” function in R to observe the trend in the annual average RSV. A cosinor model was applied for seasonal analysis according to Barnett’s research (19). Boxplots of the seasonal variation for different countries were plotted by the “season” package in R. A p-value < 0.05 was considered statistically significant.

### Ethical Requirements

This study did not involve animal experiments or clinical trials. Thus, permission from the ethical committee was not needed.

## Results

### Bibliometric Analysis

**Table 1** shows the 15 top articles on melanoma with the most annual citations from 2015 to 2019. Seven articles were published in 2015, three were published in 2016, three were published in 2017, and two were published in 2018 (20–34). The annual number of citations of these articles ranged from 167.0 to 485.0, with a median of 212.6 (170.8, 283.5). Seven of the articles were published in the *New England Journal of Medicine*, followed by *Science* (n = 4), *Cell (*n = 2), *Nature* (n = 1), and *Lancet Oncolog*y (n=1). All of the articles were original articles. These articles were then classified into three different research focuses: immunotherapy (n = 8), omics (n = 5), and microbiome (n = 2).

**Table 1:** List of the top 15 most frequently cited articles on melanoma from 2015 to 2019.

Title	Year of publication	Article type	Research focus	Journal of publication	Total citations	Annual citations	Rank by annual citations
Nivolumab in Previously Untreated Melanoma without BRAF Mutation	2015	Original article	Immunotherapy	New England Journal of Medicine	2910	485	1
Pembrolizumab versus Ipilimumab in Advanced Melanoma	2015	Original article	Immunotherapy	New England Journal of Medicine	2783	463.83	2
Gut Microbiome Modulates Response to Anti-PD-1 Immunotherapy in Melanoma Patients	2018	Original article	Microbiome	Science	888	296	3
Overall Survival with Combined Nivolumab and Ipilimumab in Advanced Melanoma	2017	Original article	Immunotherapy	New England Journal of Medicine	1134	283.5	4
Nivolumab and Ipilimumab versus Ipilimumab in Untreated Melanoma	2015	Original article	Immunotherapy	New England Journal of Medicine	1618	269.67	5
Nivolumab versus Chemotherapy in Patients with Advanced Melanoma Who Progressed after Anti-CTLA-4 Treatment (CheckMate 037): a Randomised, Controlled, Open-label, Phase 3 trial	2015	Original article	Immunotherapy	Lancet Oncology	1474	245.67	6
Improved Overall Survival in Melanoma with Combined Dabrafenib and Trametinib	2015	Original article	Immunotherapy	New England Journal of Medicine	1277	212.83	7
Mutations Associated with Acquired Resistance to PD-1 Blockade in Melanoma	2016	Original article	Omics	New England Journal of Medicine	1063	212.6	8
An Immunogenic Personal Neoantigen Vaccine for Patients with Melanoma	2017	Original article	Immunotherapy	Nature	752	188	9
The Commensal Microbiome is Associated with Anti-PD-1 Efficacy in Metastatic Melanoma Patients	2018	Original article	Microbiome	Science	558	186	10
Genomic Classification of Cutaneous Melanoma	2015	Original article	Omics	Cell	1110	185	11
Genomic and Transcriptomic Features of Response to Anti-PD-1 Therapy in Metastatic Melanoma	2016	Original article	Omics	Cell	854	170.8	12
Adjuvant Nivolumab versus Ipilimumab in Resected Stage III or IV Melanoma	2017	Original article	Immunotherapy	New England Journal of Medicine	679	169.75	13
Genomic Correlates of Response to CTLA-4 Blockade in Metastatic Melanoma	2015	Original article	Omics	Science	1005	167.5	14
Dissecting the Multicellular Ecosystem of Metastatic Melanoma by Single-cell RNA-seq	2016	Original article	Omics	Science	835	167	15

### Annual trends and seasonal variation in Google Trends

**Figure 1:**
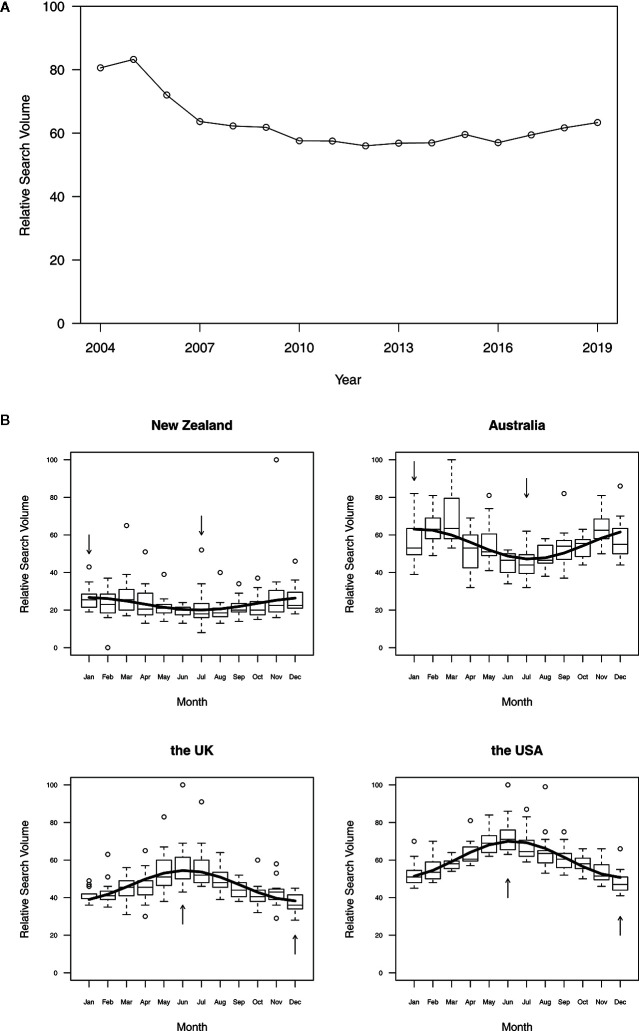
Annual trends **(A)** and seasonal variation **(B)** of the relative search volume on melanoma. a. Annual trends from 2004 to 2019. **(B)** Seasonal variation in New Zealand, Australia, the United Kingdom, and the United States. **(A)** The lines represent the overall trend of RSV variation, and the circles represent the data points of the 12-month average RSV for each year. **(B)** The seasonal analysis was conducted and fit by the cosinor model with a p-value < 0.05. The arrows indicate the extreme value of the 16-year average RSV. (Box: interquartile range (IQR). The horizontal line inside each box: median. Whisker: maximum and minimum within median ± 1.5 × IQR. Circle: outlier outside 1.5 IQR.)

The annual trends for the RSV of melanoma in Google Trends are shown in **Figure 1A**. The maximum value appeared in June 2005, and the minimum value was observed in December 2012. The average RSV remained relatively stable across the years. The seasonal variation curve fit with the “cosinor” model for the RSV is shown in **Figure 1B** (p-value < 0.05). The analysis revealed that the peak RSV of melanoma occurred in summer (January for Australia and New Zealand and June for the United States and the United Kingdom) and the valley occurred in winter (July for Australia and New Zealand and December for the United States and the United Kingdom).

### Related Topics

Topics related to melanoma from 2004 to 2019 are summarized in **Table 2**. Regarding the top related topics, “Skin” was the most related (RSV = 100), followed by “Skin cancer” (RSV = 70), “Metastasis” (RSV = 34), “Melanocytic nevus” (RSV = 32), “Nevus” (RSV = 25), “Basal-cell carcinoma” (RSV = 16), “Prognosis” (RSV = 11), “Squamous cell carcinoma” (RSV = 10), and others. Melanoma mostly originates from the skin and represents a crucial kind of metastatic skin cancer that has a poor prognosis and is difficult to distinguish from benign melanocytic nevus or other metastatic lesions, including basal cell carcinoma and squamous cell carcinoma. Regarding the rising related topics, pathological genes and monoclonal antibodies, including “BRAF,” “Ipilimumab,” “Nivolumab,” “Pembrolizumab,” and “Vemurafenib,” exhibited an increase over 5,000%, followed by associated diseases, including the topics “Squamous cell carcinoma” (n = 500%), “Basal-cell carcinoma” (n = 400%), “Melanocytic nevus” (n = 350%), and “Nevus” (n = 250%). Prognosis factors, including “Cancer staging” (n = 500%), “Metastasis” (n = 170%), “Malignancy” (n = 150%) and “Survival rate” (n = 110%), also attracted attention.

**Table 2:** Top related and rising related topics on melanoma from 2004 to 2019.

Top related topics	Relative search volume	Rising related topics	Fold changes
Skin	100	BRAF	Breakout*
Skin cancer	70	Ipilimumab	Breakout*
Metastasis	34	Nivolumab	Breakout*
Melanocytic nevus	32	Pembrolizumab	Breakout*
Nevus	25	Vemurafenib	Breakout*
Basal-cell carcinoma	16	Squamous cell carcinoma	500%
Prognosis	11	Cancer staging	500%
Squamous cell carcinoma	10	Basal-cell carcinoma	400%
Survival rate	8	Melanocytic nevus	350%
Carcinoma	7	Nevus	250%
Malignancy	7	Skin	250%
Cancer staging	7	Carcinoma	200%
Melanin	7	Skin cancer	190%
BRAF	5	Metastasis	170%
Ipilimumab	3	Malignancy	150%
Nivolumab	3	Prognosis	120%
Pembrolizumab	2	Survival rate	110%
Vemurafenib	2		

*Breakout means an increase of over 5000%.

### Annual Related Topics

The annual related topics are also compared in **Table 3** to identify the trends of the public interest over time. The top related topics each year were consistent with the above results. “Skin,” “Skin cancer,” “Metastasis,” and “Melanocytic nevus” were the only four top related topics during the 16-year interval that had nearly stable ranks, which reflected the search habits of the population. In contrast, 36 rising related topics during this period were identified and showed different emphases across the years. To facilitate comprehension, we summarized the frequency of occurrence and then classified them into several subgroups.

**Table 3:** Annual topics related to melanoma from 2004 to 2019.

Year	Top related topics	Relative search volume	Rising related topics	Fold Changes
2004	Skin	100	Basal-cell carcinoma	Breakout*
	Skin cancer	73	Melanin	Breakout*
	Metastasis	28	Prognosis	Breakout*
2005	Skin	100	Birthmark	Breakout*
	Skin cancer	79	Kaposi’s sarcoma	Breakout*
	Metastasis	30	Lymphadenectomy	Breakout*
2006	Skin	100	Melanosis	Breakout*
	Skin cancer	84	Dacarbazine	160%
	Melanocytic nevus	30	American Joint Committee on Cancer	160%
2007	Skin	100	American Joint Committee on Cancer	Breakout*
	Skin cancer	80	Dermatoscopy	Breakout*
	Metastasis	34	Freckle	250%
2008	Skin	100	Sarcoma	200%
	Skin cancer	71	Immunotherapy	180%
	Metastasis	30	Survival rate	90%
2009	Skin	100	BRAF	300%
	Skin cancer	70	Sun tanning	130%
	Metastasis	37	Dermatoscopy	120%
2010	Skin	100	Ipilimumab	400%
	Skin cancer	74	Freckle	200%
	Metastasis	33	BRAF	180%
2011	Skin	100	Melancholia	Breakout*
	Skin cancer	74	Vemurafenib	170%
	Metastasis	35	Lentigo	90%
2012	Skin	100	Mohs surgery	120%
	Skin cancer	68	Melanosis	60%
	Metastasis	36	Liver spot	60%
2013	Skin	100	Programmed cell death protein 1	300%
	Skin cancer	70	Dermatoscopy	60%
	Metastasis	34	Cell culture	60%
2014	Skin	100	Pembrolizumab	350%
	Skin cancer	70	Nivolumab	180%
	Metastasis	35	Immunotherapy	120%
2015	Skin	100	Bob Marley	150%
	Skin cancer	81	Nivolumab	150%
	Metastasis	34	Pembrolizumab	120%
2016	Skin	100	Immunotherapy	70%
	Skin cancer	75	Liver spot	50%
	Metastasis	35	Dermatoscopy	50%
2017	Skin	100	American Joint Committee on Cancer	100%
	Skin cancer	62	Melasma	90%
	Melanocytic nevus	32	Exeresis	70%
2018	Skin	100	Subungual hematoma	50%
	Skin cancer	66	Relapse	50%
	Metastasis	33	Eye neoplasm	50%
2019	Skin	100	Vulvar cancer	90%
	Skin cancer	62	Stadion	40%
	Metastasis	28	Dysplastic nevus	40%

*Breakout means an increase of over 5000%.

The diseases associated with or manifestations of melanoma appeared most frequently (17/48, 35.4%), including the terms “Freckle,” “Liver spot,” and “Melanosis” (2/48, 4.2%), followed by “Basal-cell carcinoma,” “Birthmark,” “Dysplastic nevus,” “Eye neoplasm,” “Kaposi’s sarcoma,” “Lentigo,” “Melancholia,” “Melasma,” “Sarcoma,” “Subungual hematoma,” and “Vulvar cancer” (1/48, 2.1%). Treatment options (13/48, 27.1%) included “Immunotherapy” (3/48, 6.3%), “Nivolumab,” “Pembrolizumab” (2/48, 4.2%), “Dacarbazine,” “Exeresis,” “Ipilimumab,” “Lymphadenectomy,” “Mohs surgery,” and “Vemurafenib” (1/48, 2.1%). Risk factors (5/48, 10.4%), such as the terms “BRAF” (2/48, 4.2%), “Programmed cell death protein 1,” “Sun tanning,” and “Melanin” (1/48, 2.1%), also attracted attention. Diagnostic tools (5/48, 10.4%) and prognosis (3/48, 6.3%) of melanoma, such as “Dermatoscopy” (4/48, 8.3%), “Cell culture,” “Relapse,” “Prognosis,” and “Survival rate” (1/48, 2.1%), also accounted for small portions of the annual rising related topics. Other topics (5/48, 10.4%) included the “American Joint Committee on Cancer” (3/48, 6.3%); and “Bob Marley” (1/48, 2.1%), who was a celebrity who died of melanoma; and “Stadion” (1/48, 2.1%), which had little relationship with melanoma.

## Discussion

This study updated the topics of research interest and public interest related to melanoma and provided physicians and investigators with a detailed description of the hot issues in which scientists and the public are interested. Google Trends data are a powerful tool to monitor and evaluate public interest in melanoma. The combination of Google Trends and bibliometric analysis may allow researchers to better anticipate research interests to serve melanoma patients.

Using bibliometric analysis, we determined the 15 most frequently cited articles on melanoma with the high numbers of annual citations published from 2015 to 2019. Using annual citations instead of the total citations as bibliometric parameters for ranking yielded benefits because this ranking included newly published articles that can provide emerging insights in the analysis (35). Our analysis indicated that the majority of these articles were published in the *New England Journal of Medicine*, followed by *Science, Cell*, *Nature*, and *Lancet Oncolog*y, which could be attributed to the high quality of these journals or the inherent bias with which researchers tend to select high impact factor journals for citations (36, 37). All the publications were original articles, reflecting the substantial demand of the community for revolutionary innovation and discoveries related to melanoma. The average numbers of citations of these most frequently cited articles were dramatically higher than those of other bibliometric analysis studies, such as those on rosacea (8), oral lichen planus (38), or psoriatic arthritis (38). This phenomenon reflects a high degree of research interest regarding melanoma. In addition, the articles were all classic with more than 400 citations, even for the articles published in 2018, showing the impact of the literature (8, 39).

Eight of the 15 annual most frequently cited articles were about immunotherapies, such as anti-PD1 therapies (33), nivolumab, or ipilimumab treatment (25), and nivolumab treatment in patients without BRAF mutations (27). The molecular mechanisms and the star genes that the immunotherapeutic drugs targeted, including the “Programmed cell death protein 1” (PD-1) and “B-Raf proto-oncogene” (BRAF), generated research interest (40–42). PD-1 is an immune checkpoint molecule expressed on tumor cells that inhibits CD8+ T cells and induces adaptive immune inhibition (43). PD-1 inhibitors, including “Nivolumab” and “Pembrolizumab,” have been demonstrated to show clinical activities in melanoma (44). BRAF mutations were found in approximately 60% of melanomas (45), and the inhibitors “Vemurafenib” and “Dabrafenib” were proven to be efficient in melanoma patients with the mutation (46, 47).

Furthermore, researchers might focus on other topics to provide new insights into melanoma that the public might not know. Examples include omics analysis and microbiome analysis. Genomic studies have identified activating driver mutations that stimulate the development of targeted therapies for patients (48). The overall mutational load, neoantigen load, and expression of cytolytic markers in the immune microenvironment were significantly associated with clinical benefits (29). In addition, the commensal microbiome might have a mechanistic impact on antitumor immunity in melanoma patients (23). The results suggested that patients with a favorable gut microbiome might express enhanced systemic and antitumor immunity (21).

Google Trends was particularly helpful in monitoring health information-seeking behavior and analyzing public interest. The results showed that the global average RSV for melanoma was relatively stable across the years, illustrating the continued attention given by the public to melanoma (49). Regarding seasonal analysis, in Australia and New Zealand, the peak RSV appeared in January (summer). During that time, the incidence of melanoma is predominantly high in those countries (50), and previous research has demonstrated the correlation between the RSV of sun tanning and melanoma (51). Risk factors for melanoma, including exposure to sunshine, lighter clothing, and even sun tanning, might be responsible for this result (52, 53). The health prevention campaign in Australia also promisingly reduced the rates of indoor tanning among young adults and thus helped to decrease the incidence (54). For countries in the Northern Hemisphere, such as the United States and the United Kingdom, the peak RSV appeared in June (summer), and the educational campaign of public awareness month for skin cancers in May might be responsible for increasing the RSV (55).

The related topics illustrated the most concerning themes for the public. The top related topics were defined as the most frequently searched topics within the chosen category, time, or country. As a type of cancer, melanoma mostly originates from the skin; the terms “Skin,” “Skin cancer,” and “Metastasis” were reasonably ranked in the top 3 related topics. The differential diagnosis of melanoma from other diseases such as “Melanocytic nevus” and “nevus” also attracted attention. Even senior dermatologists had some difficulties in recognizing malignant features to distinguish melanoma from nevus in dermoscopic images (56), and the involvement of artificial intelligence in dermatology liberated dermatologists and made some contributions to solving the problem (57). The terms “Basal cell carcinoma” and “Squamous cell carcinoma” refer to common malignant tumors in the United States and hence have become hot topics (58). “Malignancy,” “Prognosis,” “Relapse,” and “Survival rate” might be the most concerning topics for the patients and appeared in the list.

The rising related topics are of newly emerged public interest. The results marked “Breakout” represent tremendous increases of over 5,000% compared with the previous search, probably representing the rapid development of these topics. Immunotherapies are in the spotlight in this era. The systemic treatment of melanoma has completely changed since the first introduction of ipilimumab in 2011 (59). In less than 10 years, over 10 drugs have been proven or are being proven effective for treating unresectable melanoma and dramatically increase the predicted survival time of patients (60). A review recently summarized the historically published articles and guided clinicians regarding the use of systemic therapy for melanoma (40). The overall success explained the emergence of the public interest in immunotherapies in recent years. “Cancer staging,” “Metastasis,” “Malignancy,” and “Survival rate” also attracted attention. The complete revolution of melanoma management has invigorated the public interest in the prognoses of patients. The popularization of the concept of personalized medicine caused the public to become more concerned with the outcomes of patients instead of short-term effects. Hence, it was necessary to formulate an individualized systemic medication plan according to the cancer stage and metastasis of the patients to achieve the maximum survival rate.

The annual top related topics were analyzed to reveal the trends in the topics of greatest interest during 2004 to 2019. Most of these topics were consistent with the above discussion, but some interesting terms also emerged. “Basal-cell carcinoma,” “Birthmark,” “Dysplastic nevus,” “Eye neoplasm,” “Freckle,” “Kaposi’s sarcoma,” “Liver spot,” “Lentigo,” “Melancholia,” “Melanosis,” “Melasma,” “Sarcoma,” “Subungual hematoma,” and “Vulvar cancer” were the diseases associated with or manifestations of melanoma (61–63). Ocular melanoma is the second most common type of melanoma and is often observed as an eye neoplasm. Lentigo maligna might eventually develop into invasive melanoma (64). “Melancholia,” “Melanosis,” and “Melasma” might have similar spellings as melanoma and hence confuse the searchers.

Treatment methods ranked second among the results. Terms associated with surgical methods including “Exeresis” and “Mohs surgery” refer to effective treatment modalities for early-stage noninvasive melanoma and therefore attract public interest (65, 66). Consistent with the bibliometric analysis, immunotherapies and risk genes attracted attention. In addition to those we discussed above, CTLA-4 was recently the focus of the public and appeared on the list. CTLA-4 is an immune checkpoint molecule that downregulates pathways of T cell activation (67), and “Ipilimumab” can inhibit CTLA-4 to improve survival in patients with metastatic melanoma (68).

Risk factors that had been discussed above, including sun tanning and melanin, illustrated the importance of public educational campaigns (69, 70). The evolution and broad adaption of dermatoscopy in clinical examinations also improved the diagnosis of benign and malignant cutaneous neoplasms compared with diagnosis with unaided eyes. Dermatoscopy also improved the ability of expert readers to make appropriate management decisions (71). Cell cultures can contribute to the diagnosis and development of melanoma management plans and function as an experimental tool to facilitate the development of new drugs (72). Interestingly, American Joint Committee on Cancer and a celebrity, Bob Marley, who died of the disease, also appeared on the list. The former association formulates the guidelines for the cancer staging of melanoma, and the latter reflects the celebrity effect, which can stimulate the recognition of the disease among the public.

Our study revealed the consistency between the research interest and the public interest. Both interests focused on the risk genes of melanoma and their inhibitors or blockers. These included PD-1, BRAF, CTLA-4, ipilimumab, nivolumab, dabrafenib, and trametinib. The use of social media has substantially increased among researchers and the public and could explain this corresponding relationship (73). In Australia, the SunSmart skin cancer prevention program has been demonstrated to contribute to the reduction of melanoma among younger cohorts (74). In addition to Australia, the Euromelanoma campaign also organized a yearly media campaign, which targets the public and focuses on different aspects of melanoma prevention. Euromelanoma Day has been held each year in May, both in university‐based and hospital‐based outpatient clinics and private dermatology surgeries (75). Patients and even the normal population can enhance their knowledge through these campaigns and become familiar with the latest research interest (76). In addition, the research interest might be influenced by social media, as reported by Pemmaraju (74), and the types of tweets about skin cancer have changed rapidly over time. The number of pharmaceutical companies that is discussed has been increasing, and the topic tags transitioned from “melanoma” to “immunotherapies” from 2011 to 2016 (74).

However, some differences still exist. The public did not show interest in the omics and microbiomes of melanoma that the research community studied. This was comprehensive because the public might not be familiar with these academic terms. More importantly, patients were mostly concerned with the symptoms, differential diagnosis, metastasis, and treatment of melanoma, especially newly emerged targeted drugs, which might improve prognosis and predict survival time. These aspects might become future directions for research and the popularization of science. Mechanisms, pathogenesis, pathophysiology, and epidemiological features were probably less important for patients because the complete elucidation of such factors could not alleviate symptoms, cure the disease, and decrease the high treatment expenses. Although these research fields might not provide patients and their families with hope in this era, they remain valuable for researchers. The development of new techniques and the discovery of key molecules in melanoma are crucial to guide future management. The prognosis of melanoma patients with regional metastases is influenced by the genomic classification, offering insights to further personalize therapeutic decision making (20). In addition, the commensal microbiome might have a mechanistic impact on antitumor immunity in melanoma patients (23). Such research findings might be included in educational campaigns in the future.

There are several limitations to the study. First, the public interest is restricted to Internet users who are conducting Google searches in English. There may be selection bias because the disease might not attract enough attention in underdeveloped areas. Although English remains the most popular official language worldwide, different languages and cultures could have different interests. In addition, other search engines could also be more popular than Google Trends in certain countries. For example, the Baidu engine is the main search engine in China. To compensate for the loss of data, we tried to use “topics” instead of “terms” as keywords, which may include some synonyms of melanoma in other languages. Second, only the Web of Science database was used to search for eligible articles, and some articles may be missed. Notably, fewer citations do not mean that an article is unimportant because it may lack the ability to be accessed by scholars.

## Conclusion

This study used bibliometric and Google Trends analyses to update the topics and to compare the differences and similarities of research interest and public interest in melanoma. Regarding research interest, the top 15 most frequently cited articles each year focused on immunotherapy (n=8), omics (n=5), and the microbiome (n=2). Regarding public interest, diseases associated with or manifestations of melanoma, treatment options, risk factors, diagnostic tools, and prognosis were of the greatest interest to the public. The results revealed the trends in research interest and public interest in melanoma, which may pave the way for further research.

## Data Availability Statement

The original contributions presented in the study are included in the article/supplementary material. Further inquiries can be directed to the corresponding author.

## Author Contributions

HZ and QS conceived and designed the study. HZ and YZW prepared the manuscript, and had equal contribution to the study. QZ and KT prepared the tables and figures. QZ, KT, RF, YCW, and QS reviewed and revised the manuscript. All authors contributed to the article and approved the submitted version.

## Funding

The publishing fee was funded by Peking Union Medical College Hospital.

## Conflict of Interest

The authors declare that the research was conducted in the absence of any commercial or financial relationships that could be construed as a potential conflict of interest.
